# Assessing risk for Mendelian disorders in a Bronx population

**DOI:** 10.1002/mgg3.307

**Published:** 2017-07-06

**Authors:** Guy diSibio, Kinnari Upadhyay, Philip Meyer, Carole Oddoux, Harry Ostrer

**Affiliations:** ^1^ Department of Clinical Science California Northstate University College of Medicine Elk Grove California; ^2^ Department of Pathology Albert Einstein College of Medicine Bronx New York

**Keywords:** Carrier screening, genetic testing, variant annotation, whole genome sequencing

## Abstract

**Background:**

To identify variants likely responsible for Mendelian disorders among the three major ethnic groups in the Bronx that might be useful to include in genetic screening panels or whole exome sequencing filters and to estimate their likely prevalence in these populations.

**Methods:**

Variants from a high‐density oligonucleotide screen of 192 members from each of the three ethnic‐national populations (African Americans, Puerto Ricans, and Dominicans) were evaluated for overlap with next generation sequencing data. Variants were curated manually for clinical validity and utility using the American College of Medical Genetics (ACMG) scoring system. Additional variants were identified through literature review.

**Results:**

A panel of 75 variants displaying autosomal dominant, autosomal recessive, autosomal recessive/digenic recessive, X‐linked recessive, and X‐linked dominant inheritance patterns representing 39 Mendelian disorders were identified among these populations.

**Conclusion:**

Screening for a broader range of disorders could offer the benefits of early or presymptomatic diagnosis and reproductive choice.

## Introduction

Ethnicity‐based genetic testing, particularly among well‐defined ethnic groups, has been advocated as a valuable strategy that can aid in preventing genetic disease and improving public health at relatively low cost (Strauss et al. 2012).The advantage of such population‐targeted screening has included the ability to identify carriers of well‐characterized, population‐associated deleterious recessive and X‐linked conditions and thereby prevent the birth of affected children. One notable success of this approach has been the sharp decline in incidence of Tay‐Sachs disease and other disorders among Ashkenazi Jews following carrier screening for this condition (Kaback 2000). Yet despite these successes, there are indications that the spectrum of population‐based risk for Mendelian disorders has been underestimated. For example, a recent study documenting carrier frequencies for 108 high‐penetrance autosomal recessive conditions among individuals from 14 ethnic groups has demonstrated population risks beyond the original populations known to be at risk for these conditions (Lazarin et al. 2013). Genome wide approaches to assess population risk for Mendelian conditions can further expand our understanding of the spectrum of disorders for which specific populations may be at risk. Together with the availability of highly multiplexed technologies, such as high density oligonucleotide arrays, this information can be used to develop more comprehensive, cost effective diagnostic and reproductive risk assessment assays. In addition, it enables the incorporation of predisposition and presymptomatic testing, such as for cancer risk.

Of the ethnic groups polled by the US Census Bureau, the largest U.S. ethnic groups are African Americans and Hispanic Latinos, comprising 13.3% and 17.6% of the population, respectively (https://www.census.gov/quickfacts/table/PST045216/00‐ accessed 3/16/17). Population growth of Hispanic Latinos accounted for over half of the growth of the total US population between 2000 and 2010. Puerto Ricans and Dominicans comprise 9.2% and 2.2% respectively of the total Hispanic Latino population and are concentrated in the major urban cities of New York, Florida, New Jersey, Pennsylvania, Massachusetts, California and Illinois (http://www.census.gov/prod/cen2010/briefs/c2010br-04.pdf accessed 3/16/17 [PDF]) making them important populations in multiple regions of the US.

Although Puerto Ricans and Dominicans are products of admixture from similar source populations (Arawak Native American, West and Central African, and Iberian European), differences in the relative source population proportions, time of contact, number of contact events, and population growth after isolation have led to the development of genetically distinct populations with individual founder mutations (Li et al. 2014). Despite Sewall Wright's papers about island populations developing unique genetic constitutions based on migration, gene flow, selection and drift beginning as early as 1931 (Berniell‐Lee et al. 2009), the genetic distinctiveness of these island populations has only recently been appreciated and as a result most researchers, clinicians, and population databases have used only the broad classification of Hispanic Latino. Thus our understanding of the clinically relevant genetic risks in these populations remains incomplete, limiting the utility of genetic testing among these populations. In addition, suboptimal provider communication, medical mistrust, and cultural beliefs, have further limited access to testing and understanding of genetic risks (Lalueza‐Fox et al. 2001; Burke et al. 2006; Kaplan et al. 2006; Suther and Kiros 2009; Burke and Korngiebel 2015).

In the borough of Bronx, New York, African Americans and two Hispanic Latino subgroups (Puerto Ricans and Dominicans) collectively comprise 85% of the population of 1.4 million people (http://www.census.gov accessed 5‐26‐11). As a first step in developing better genetics services for these groups, we sought to expand our understanding of the deleterious genetic variants that they carry by screening 192 healthy individuals from each of these three ethno national groups using Affymetrix Axiom 319 arrays. The more than 300,000 probes found on Affymetrix Axiom 319 arrays are a group of markers enriched for likely pathogenic variants ascertained by whole exome screening of 120,000 ethnically diverse samples of European, African, Latino, and Asian ancestry representing multiple disease cohorts including type 2 diabetes, cancer, infectious disease, cardiovascular disease, and neurological/psychiatric disorders. Our study screens Dominicans, Puerto Ricans, and African Americans, to determine whether any of these variants are relevant to these specific populations. Detected variants are further confirmed to be present in these specific populations and their frequency estimated by comparison to population‐specific exome sequencing data sets. Confirmed variants are annotated according to ACMG guidelines (Richards et al. 2015) to provide a working set of common genetic disease risk variants useful to clinicians evaluating patients from these populations. Parallel literature review was also used to identify additional conditions and underlying variants documented for our populations of interest.

The parallel examination of these three groups offers distinct advantages. First, their overlapping Columbian and post‐Columbian histories and occupancy of a small urban area suggest gene flow and consequent shared health risk, while their divergent pre‐Columbian histories predict some disease risks unique to each group (Lalueza‐Fox et al. 2001; Berniell‐Lee et al. 2009; Li et al. 2014). Second, despite residing in New York, an area of potentially sophisticated medical knowledge and intervention, these populations continue to experience rates of morbidity and mortality from diseases with substantial genetic underpinnings similar to those seen among underserved populations throughout the U.S. (Kaplan et al. 2006). Studies, such as ours, might ultimately engender broader use of and trust for genetic screening among these groups, providing a step toward closing public healthcare gaps (Burke et al. 2006; Suther and Kiros 2009; Burke and Korngiebel 2015).

## Materials and Methods

### Ethical compliance

Patients were consented for anonymous use of residual specimen post clinical testing according to an NYU IRB‐approved protocol.

Blood samples were drawn from 192 individuals from each of the Bronx's three prominent ethnonational groups: Puerto Ricans, African Americans, and Dominicans. The subjects studied were healthy unrelated individuals (predominantly female) presenting for routine reproductive screening and reproductive risk assessment. Ethnonationality was self‐reported as parental country of origin at the time of collection. Each subject's DNA was extracted through a standard protocol and hybridized to a separate Affymetrix Axiom^®^ Exome 319 Genotyping array containing 318,916 probes using a similar experimental design to that reported previously (Cartmel et al. 2014). All samples genotype calling were performed using Affymetrix Genotyping Console^TM^ and following a standardized best practices protocol. Variants excluded from further analysis included those with mean allele frequencies (MAF) of either 0 (not present) or 1 (homozygous), and those that did not meet the expectations of Hardy‐Weinberg equilibrium (*P*‐value <0.05).

**Figure 1:**
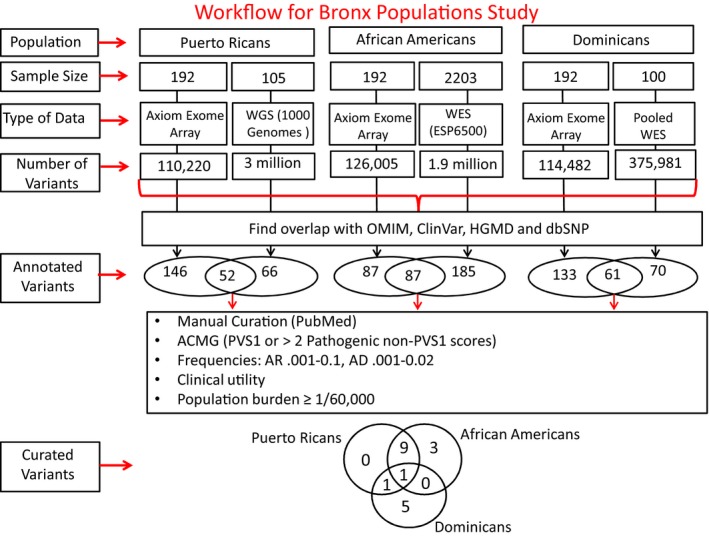
Workflow for identification of variants listed in Table 1 (see Materials and Methods for description). For Puerto Ricans and African Americans, the number of variants identified through Axiom Exome Array is designated along the left vertical arrow, the number identified through 1000 Genomes and/or ESP6500 along the right vertical arrow, and overlaps following subsequent pipeline steps as the intersection between ovals. For Dominicans, the number of variants identified through Axiom Exome Array is designated along the left vertical arrow, the number identified through pooled whole exome sequencing along the right vertical arrow, and overlaps as the as the intersection between the two ovals.

In parallel, DNA samples from 100 Dominican subjects, randomly selected from our 192 sample set, were combined into 10 pools comprising 20 specimens each and subjected to whole exome sequencing (WES) on an Illumina platform. This strategy ensured that each exome was represented in two separate pools and thereby sequenced twice to ensure fidelity (Prabhu and Pe'er 2009). Variant calling was performed following a method used previously in our laboratory (Isakov et al. 2013). The pooled WES served as orthogonal validation for variants identified through the Axiom 319 chip in Dominicans (see Fig. 1). Orthogonal validation was also performed using the 1000 Genomes and ESP6500 databases for Puerto Ricans and African Americans, respectively (Genomes Project Consortium, 2010; Tennessen et al. 2012).

Variants were parsed by a custom web‐crawler that queried three interrelated, open‐source databases for pathogenicity: OMIM (http://omim.org), ClinVar (https://www.ncbi.nlm.nih.gov/clinvar/), and dbSNP (http://www.ncbi.nlm.nih.gov/SNP/) (Baskovich et al. 2016). Variants flagged in the pipeline as pathogenic or possibly pathogenic were evaluated by at least two independent reviewers who performed literature review to confirm or reject the assessment. To compile the final variant list, variants were assigned one or more scores from the variant calling guidelines of the ACMG (Richards et al. 2015). To augment the panel, the literature was queried for variants underlying conditions known to be present in these populations of interest which were subjected to a similar approach for assessing pathogenicity.

Estimated carrier frequencies and disease burden were calculated assuming Hardy‐Weinberg equilibrium and using a Mean Minor Allele Frequency (MMAF) that weighted individual frequencies based on the number of subjects contributed by the respective database to the entire cohort. For Puerto Ricans, 1000GenomesPUR contributed 210 (39%) and Axiom 319 contributed 384 (61%) of all alleles to the final allele frequency pool. For African Americans, ESP6500 contributed 4406 (90%), 1000GenomesASW contributed 132 (3%), and Axiom 319 contributed 384 (7%) of all alleles to the final allele frequency pool. For Dominicans, whole exome sequencing contributed 200 (34%) and Axiom 319 contributed 384 (66%) of all alleles to the final allele frequency pool.

“At risk” variants are reported for a likely disease burden of 1/60,000 or greater, as in our previous study (Baskovich et al. 2016). Thus, inclusion in the panel was limited to alleles with a MMAF of 0.001–0.1 for autosomal recessive or X‐linked conditions and 0.001–0.02 for autosomal dominant conditions (Cartmel et al. 2014). Conditions in the panel that exceeded these limits included *APOL1*NM_003661.3:c.1152T>G(p.Ile384Met) and NM_003661.3:c.1024A>G(p.Ser342Gly) and *G6PD* NM_000402.4:c.292G>A(p.Val68Met), all of which have been hypothesized to have increased in frequency in ancestral African populations because of heterozygote advantage (Genovese et al. 2010; Sirugo et al. 2014). The panel also included alleles for some conditions with rare prevalence among one of these populations for which testing would have clinical utility.

## Results

This study identified 75 variants for 39 conditions, including seven autosomal dominant, 29 autosomal recessive, one autosomal recessive/digenic recessive, one X‐linked recessive, and one X‐linked dominant inheritance) (Tables 1, S1 and S2). Of these conditions, 10 were observed in this study or reported previously for African Americans and Puerto Ricans, two were observed in this study or reported previously for African Americans and Dominicans and three were observed in this study or reported previously for all three populations. Some of the early‐onset autosomal recessive conditions in the panel are included in current newborn screening panels, such as galactosemia, cystic fibrosis, hemoglobin S and hemoglobin C disease (Watson et al. 2006). Some of these, such as cystic fibrosis, SMA and fragile X syndrome, are included in panels recommended for heterozygote detection (Grody et al. 2013). The remainder has not been recommended for population‐based testing.

**Table 1:** List of all conditions identified in this study by inheritance pattern in Puerto Ricans (PR), African Americans (AA), Dominicans (Dom). Shaded: Conditions identified by literature review. Unshaded: Conditions identified through the pipeline

Phenotype	HPO#	Inheritance pattern	Populations at Risk		
			PR	AA	Dom
Autosomal dominant (*n* = 7)					
Amyloidosis, hereditary, transthyretin‐related	HP:0011034	AD	Y	Y	
Bone marrow failure, telomere‐related	HP:0005528	AD		Y	
Breast‐ovarian cancer, familial 1	HP:0003002	AD	Y	Y	
Breast‐ovarian cancer, familial 2	HP:0003002	AD	Y		
Charcot‐Marie‐tooth disease, familial 2A2	HP:0002460	AD	Y	Y	
Retinitis pigmentosa 1	HP:0001133	AD	Y	Y	
Retinitis pigmentosa 35	HP:0001133	AD	Y	Y	
Autosomal recessive (*n* = 29)					
Alkaptounria	HP:0000079	AR			Y
Alpha thalassemia	HP:0001878	AR		Y	
Alpha‐1‐antitrypsin deficiency	HP:0002910	AR	Y	Y	
Beta thalassemia	HP:0001871	AR		Y	
Cystic fibrosis	HP:0001738	AR		Y	
Deafness, autosomal recessive	HP:0000365	AR		Y	Y
Dystonia, DOPA‐responsive	HP:0001332	AR			Y
Focal segmental glomerulosclerosis 4, susceptibility to	HP:0000097	AR	Y	Y	
Galactosemia	HP:0012024	AR		Y	
Gaucher disease	HP:0002172	AR		Y	
Glycogen storage disease X	HP:0003546	AR		Y	
Hemochromatosis	HP:0001402	AR	Y	Y	Y
Hemoglobin C disease	HP:0001871	AR	Y	Y	
Hereditary folate malabsorption	HP:0004851	AR	Y		
Hermansky‐Pudlak syndrome 1	HP:0001107	AR	Y		
Hermansky‐Pudlak syndrome 3	HP:0001107	AR	Y		
Hypogonadotrophic hypogonadism 7, without anosmia	HP:0000044	AR		Y	Y
Ichthyosis, congenital, autosomal recessive 1	HP:0007479	AR		Y	
Interleukin 1 receptor antagonist deficiency	HP:0000962	AR	Y		
Leber congenital amaurosis 6	HP:0000548	AR	Y	Y	
McArdle disease	HP:0003710	AR			Y
Muscular dystrophy, limb‐girdle, type 2L	HP:0002659	AR			Y
Prothrombin deficiency	HP:0003010	AR	Y		
Pseudovaginal perineoscrotal hypospadias	HP:0000033	AR			Y
Retinitis pigmentosa 14	HP:0000510	AR			Y
Sickle cell anemia	HP:0001871	AR	Y	Y	Y
Smith‐Lemli‐Opitz syndrome	HP:0000033	AR		Y	
Spondylocostal dysostosis 2	HP:0003422	AR	Y		
Steel syndrome	HP:0002650	AR	Y		
Autosomal recessive/digenic recessive (n=1)					
Bardet‐Biedel syndrome	HP:0000510	AR, DR	Y		
X‐linked dominant (*n* = 1)					
Fragile X syndrome	HP:0002342	XLD	Y	Y	Y
X‐linked recessive (*n* = 1)					
G6PD deficiency	HP:0004814	XLR	Y	Y	

Predominantly adult onset conditions included those inherited in both autosomal recessive and dominant patterns and whose identification might be beneficial in leading for expedited intervention and/or anticipatory monitoring. Variants conferring risk for both malignant and nonmalignant diseases are included in this category. Eight high risk variants in *BRCA1* and *BRCA2* were identified among African Americans and Puerto Ricans (Dean et al. 2015). Among the non‐malignant conditions identified in Puerto Ricans and African Americans were transthyretin amyloidosis (*TTR*NM_000371.3:c.424G>A(p.Val142Ile)) that typically manifests after age 60 with renal amyloidosis and cardiomyopathy (Jacobson et al. 1997; Shah et al. 2016) and the APOL1‐related kidney diseases (*APOL1*NM_003661.3:c.1152T>G(p.Ile384Met) and NM_003661.3:c.1024A>G(p.Ser342Gly)), focal segmental glomerulosclerosis, HIV‐associated nephropathy (HIVAN), and hypertension‐associated end stage kidney disease (ESKD) with risks significantly higher for those carrying two risk alleles (Genovese et al. 2010).

Based on a conservative estimate for medically significant conditions, for Puerto Ricans, the panel would detect 50% of individuals as heterozygotes for autosomal recessive disorders and 2.5% as heterozygotes for autosomal dominant disorders. Among couples, 3% would be at risk for having a child with a recessive disorder. For African Americans, the panel would detect 87% of individuals as heterozygotes for autosomal recessive disorders and 4.6% as heterozygotes for autosomal dominant disorders. Among couples 23% would be at risk for having a child with a recessive disorder. For Dominicans, the panel would detect 23% of individuals as heterozygotes for autosomal recessive disorders. Among couples 1.1% would be at risk for having a child with a recessive disorder.

**Figure 2:**
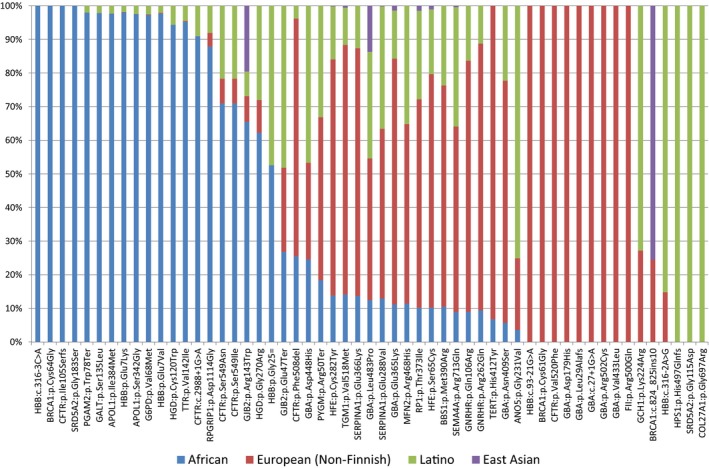
Origins of variants. Allele frequencies from the ExAC database (http://exac.broadinstitute.org) were collected to determine the likely origin of the variants from African, European (Non‐Finnish), Latino and East Asian. These allele frequencies were then normalized to each other and plotted to visualize the relative frequency of each variant.

In order to gain insights into the population origins of the clinically significant variants identified in our study, we queried their frequencies among the populations known to have admixed during the peopling of the Americas (Lalueza‐Fox et al. 2001) (Fig. 2). These populations included various European and African groups, for which there is representation in both the 1000G and ExAC databases. As no equivalent database for the pre‐existing Caribbean Indigenous/Native populations (Tarawak tribes) exists, the ExAC East Asian database was queried as a proxy for these frequencies, given the archeological record of migration through the Bering Straits in populating the New World. Several variants were found exclusively among African and/or Hispanic‐Latino populations suggesting an African origin. These included *HBB*:*NM_000518.4:c.316‐3C>A*,* BRCA1*:NM_007294.3:c.190T>G(p.Cys64Gly), *CFTR*:NM_000492.3:c.313delA(p.Ile105Serfs), *SRD5A2*:NM_000348.3:c.547G>A(p.Gly183Ser), *PGAM2*:NM_000290.3:c.233G>C(p.Trp78Ter), *GALT*:NM_000155.3:c.404C>T(p.Ser135Trp), *APOL1*:NM_003661.3:c.1152T>G(p.Ile384Met), *HBB*:NM_000518.4:c.19G>A(p.Glu7Lys), *APOL1*:NM_003661.3:c.1024A>G(p.Ser342Gly), *G6PD*:NM_000402.4:c.292G>A(p.Val68Met), *HBB*:NM_000518.4:c.20A>G(p.Glu7Ala), *HGD*:NM_000187.3:c.360T>C(p.Cys120Trp) and *TTR*:NM_000371.3:c.424G>A(p.Val142Ile). Examples of variants with relatively increased frequency among Europeans and evidence of gene flow toward African Americans and Hispanic Latinos (given their relatively lower frequencies among these populations) include *HBB*:NM_000518.4:c.93‐21G>A, *BRCA1*:NM_007294.3:c.181T>G(p.Cys61Gly), *CFTR*:NM_000492.3:c.1558G>T(p.Val520Phe), *GBA*:NM_001005741.2:c.[535G>C:c.1093G>A](p.As179His:p.Glu365Lys), *GBA*:NM_001005741.2:c.115+1G>A, *GBA*:NM_001005741.2:c.1504C>T(p.Arg502Cys), *GBA*:NM_001005741.2:c.1297G>T(p.Val433Leu), and *F2*:NM_000506.4:c.1499G>A(p.Arg500Gln). Several conditions are observed in single populations and are likely to have arisen from founder mutations. Among Puerto Ricans, these include Hermansky‐Pudlak syndrome (*HPS1*:NM_000195.4(HPS1):c.1487_1488insCCAGCAGGGGAGGCCC (p.His497Glnfs)) and Steel syndrome (*COL27A1*:NM_032888.3:c.2089G>C(p.Gly697Arg)) and, among Dominicans, this includes pseudovaginal perineal scrotal hypospadias from 5‐alpha‐reductase deficiency (*SRD5A2*:NM_000348.3:c.344G>A(p.Gly115Asp)). Variants with significant overlap with East Asian populations such as BRCA1 NM_007294.3:c.815_825dupAGCCATGTGG(p.Thr276Alafs) may have been passed to Native American populations or may be coincidental and have arisen twice, in Asia and the Americas.

## Discussion

The aim of this study was to increase understanding of the genetic disease burden carried by Puerto Ricans, African Americans, and Dominicans as a first step in developing more effective clinical diagnosis and management strategies for these populations. We have broadened the list of clinically relevant conditions beyond those traditionally associated with these populations. Screening for the recessive and X‐linked disorders provide opportunities for reproductive risk management, beyond conditions currently included in ACMG‐recommended or commercially available panels.

Early or presymptomatic identification of other conditions could lead to interventions that would reduce morbidity and mortality and in some cases can reduce the diagnostic odyssey. The features of hereditary folate malabsorption include basal ganglion calcification, mental retardation, movement disorders, seizure, and megaloblastic anemia. Recognition of this disorder and consequent folate supplementation among affected individuals has been effective in avoiding morbidities associated with HFM (Qiu et al. 2006). The increased prevalence has prompted advocacy of newborn screening for this mutation among Puerto Ricans (Mahadeo et al. 2011). Transthyretin amyloidosis presents as a late‐onset autosomal dominant disease with renal amyloidosis and cardiomyopathy and disproportionately affects African Americans (Jacobson et al. 1997; Shah et al. 2016). Liver transplant remains the only potentially curative treatment for transthyretin amyloidosis, and is best accomplished at an early age to reduce perioperative morbidity secondary to cardiac pathology. Close monitoring of renal and cardiac function may be pursued for non‐operative candidates, and correct identification may guide appropriate supportive treatment. Either scenario emphasizes the personal clinical utility of presymptomatic genetic testing.

Predicting breast cancer risk through testing *BRCA1* and *BRCA2* high‐risk variants seen in our populations represents a genetic screening paradigm shift to a broader definition of population screening to include predisposition screening (Lieberman et al. 2016). Despite an increased frequency of aggressive triple‐negative breast cancer among African American and Hispanic Latino women, genetic testing for deleterious BRCA 1 and 2 high‐risk variants to guide clinical decisions remains underutilized by members of either population (Olopade et al. 2003; Halbert et al. 2006; Lieberman et al. 2016). Yet optimistically, women from these populations who are at moderate to high risk for carrying *BRCA1* and *BRCA2* alleles might be more receptive to genetic testing (Kessler et al. 2005).

Arguments have been proposed, both in favor of and in opposition to, ethnicity‐based genetic screening of unaffected adults. Arguments in favor of such screening cite the greater ease of identification and confidence in clinical management of well understood genetic conditions and their underlying genetic alterations. Examples of this paradigm include successes in reducing morbidity from maple syrup urine disease and glutaric acidemia type 1 among Amish and Mennonite communities in Northern Pennsylvania and Tay‐Sachs disease among Ashkenazi Jews (Kaback 2000; Strauss et al. 2012). Arguments in opposition to such testing focus on concerns of potential discrimination and compulsory genetic testing for certain groups, as was the case for sickle cell disease during the 1970s (Fulda and Lykens 2006). Further, a recent study cited risk of a negative self‐image among individuals discovered to carry an autosomal recessive deleterious mutation, even when reassured of their lack of personal risk (Axworthy et al. 1996). Such concerns and the variety of disorders identified and ways in which this information is applied (self‐diagnosis or reproductive risk assessment) highlight the need for efforts to develop individualized and culturally sensitive genetic counseling to facilitate informed consent and access to the genetic services for these populations.

## Conflict of Interest

None declared.

## Supporting information


**Table S1** List of conditions identified in this study by inheritance pattern in Puerto Ricans (PR), African Americans (AA), and Dominicans (Dom), identified through the analytical pipeline.
**Table S2** List of conditions identified in this study by inheritance pattern in Puerto Ricans (PR), African Americans (AA), and Dominicans (Dom), identified through literature review.

## References

[mgg3307-bib-0001] Axworthy, D. , D. J. Brock , M. Bobrow , and T. M. Marteau . 1996 Psychological impact of population‐based carrier testing for cystic fibrosis: 3‐year follow‐up. UK Cystic Fibrosis Follow‐Up Study Group. Lancet 347:1443–1446. 8676627 10.1016/s0140-6736(96)91683-9

[mgg3307-bib-0002] Baskovich, B. , S. Hiraki , K. Upadhyay , P. Meyer , S. Carmi , N. Barzilai , et al. 2016 Expanded genetic screening panel for the Ashkenazi Jewish population. Genet. Med. 18:522–528. 26334176 10.1038/gim.2015.123 PMC4814352

[mgg3307-bib-0003] Berniell‐Lee, G. , F. Calafell , E. Bosch , E. Heyer , L. Sica , P. Mouguiama‐Daouda , et al. 2009 Genetic and demographic implications of the Bantu expansion: insights from human paternal lineages. Mol. Biol. Evol. 26:1581–1589. 19369595 10.1093/molbev/msp069

[mgg3307-bib-0004] Burke, W. , and D. M. Korngiebel . 2015 Closing the gap between knowledge and clinical application: challenges for genomic translation. PLoS Genet. 11:e1004978. 25719903 10.1371/journal.pgen.1004978 PMC4342348

[mgg3307-bib-0005] Burke, W. , M. J. Khoury , A. Stewart , R. L. Zimmern , and G. Bellagio . 2006 The path from genome‐based research to population health: development of an international public health genomics network. Genet. Med. 8:451–458. 16845279 10.1097/01.gim.0000228213.72256.8c

[mgg3307-bib-0006] Cartmel, B. , A. Dewan , L. M. Ferrucci , J. Gelernter , J. Stapleton , D. J. Leffell , et al. 2014 Novel gene identified in an exome‐wide association study of tanning dependence. Exp. Dermatol. 23:757–759. 25041255 10.1111/exd.12503 PMC4204712

[mgg3307-bib-0007] Dean, M. , J. Boland , M. Yeager , K. M. Im , L. Garland , M. Rodriguez‐Herrera , et al. 2015 Addressing health disparities in Hispanic breast cancer: accurate and inexpensive sequencing of BRCA1 and BRCA2. Gigascience 4:1–13. 26543556 10.1186/s13742-015-0088-z PMC4634732

[mgg3307-bib-0008] Fulda, K. G. , and K. Lykens . 2006 Ethical issues in predictive genetic testing: a public health perspective. J. Med. Ethics 32:143–147. 16507657 10.1136/jme.2004.010272 PMC2564466

[mgg3307-bib-0009] Genomes Project Consortium , G. R. Abecasis , D. Altshuler , A. Auton , L. D. Brooks , R. M. Durbin , R. A. Gibbs , M. E. Hurles , and G. A. Mcvean . 2010 A map of human genome variation from population‐scale sequencing. Nature 467:1061–1073. 20981092 10.1038/nature09534 PMC3042601

[mgg3307-bib-0010] Genovese, G. , D. J. Friedman , M. D. Ross , L. Lecordier , P. Uzureau , B. I. Freedman , et al. 2010 Association of trypanolytic ApoL1 variants with kidney disease in African Americans. Science 329:841–845. 20647424 10.1126/science.1193032 PMC2980843

[mgg3307-bib-0011] Grody, W. W. , B. H. Thompson , A. R. Gregg , L. H. Bean , K. G. Monaghan , A. Schneider , et al. 2013 ACMG position statement on prenatal/preconception expanded carrier screening. Genet. Med. 15:482–483. 23619275 10.1038/gim.2013.47

[mgg3307-bib-0012] Halbert, C. H. , L. Kessler , J. E. Stopfer , S. Domchek , and E. P. Wileyto . 2006 Low rates of acceptance of BRCA1 and BRCA2 test results among African American women at increased risk for hereditary breast‐ovarian cancer. Genet. Med. 8:576–582. 16980814 10.1097/01.gim.0000237719.37908.54

[mgg3307-bib-0013] Isakov, O. , E. S. Rinella , D. Olchovsky , I. Shimon , H. Ostrer , N. Shomron , et al. 2013 Missense mutation in the MEN1 gene discovered through whole exome sequencing co‐segregates with familial hyperparathyroidism. Genet. Res. 95:114–120. 10.1017/S0016672313000141 24074368

[mgg3307-bib-0014] Jacobson, D. R. , R. D. Pastore , R. Yaghoubian , I. Kane , G. Gallo , F. S. Buck , et al. 1997 Variant‐sequence transthyretin (isoleucine 122) in late‐onset cardiac amyloidosis in black Americans. N. Engl. J. Med. 336:466–473. 9017939 10.1056/NEJM199702133360703

[mgg3307-bib-0015] Kaback, M. M. 2000 Population‐based genetic screening for reproductive counseling: the Tay‐Sachs disease model. Eur. J. Pediatr. 159(Suppl 3):S192–S195. 11216898 10.1007/pl00014401

[mgg3307-bib-0016] Kaplan, S. A. , N. S. Calman , M. Golub , J. H. Davis , C. Ruddock , and J. Billings . 2006 Racial and ethnic disparities in health: a view from the South Bronx. J. Health Care Poor Underserved 17:116–127. 10.1353/hpu.2006.0026 16520520

[mgg3307-bib-0017] Kessler, L. , A. Collier , K. Brewster , C. Smith , B. Weathers , E. P. Wileyto , et al. 2005 Attitudes about genetic testing and genetic testing intentions in African American women at increased risk for hereditary breast cancer. Genet. Med. 7:230–238. 15834240 10.1097/01.gim.0000159901.98315.fe

[mgg3307-bib-0018] Lalueza‐Fox, C. , F. L. Calderon , F. Calafell , B. Morera , and J. Bertranpetit . 2001 MtDNA from extinct Tainos and the peopling of the Caribbean. Ann. Hum. Genet. 65:137–151. 11427174 10.1017/S0003480001008533

[mgg3307-bib-0019] Lazarin, G. A. , I. S. Haque , S. Nazareth , K. Iori , A. S. Patterson , J. L. Jacobson , et al. 2013 An empirical estimate of carrier frequencies for 400+ causal Mendelian variants: results from an ethnically diverse clinical sample of 23,453 individuals. Genet. Med. 15:178–186. 22975760 10.1038/gim.2012.114 PMC3908551

[mgg3307-bib-0020] Li, S. , C. Schlebusch , and M. Jakobsson . 2014 Genetic variation reveals large‐scale population expansion and migration during the expansion of Bantu‐speaking peoples. Proc. Biol. Sci. 281:pii:20141448. 10.1098/rspb.2014.1448 PMC4173682 25209939

[mgg3307-bib-0021] Lieberman, S. , A. Lahad , A. Tomer , C. Cohen , E. Levy‐Lahad , and A. Raz . 2016 Population screening for BRCA1/BRCA2 mutations: lessons from qualitative analysis of the screening experience. Genet. Med. 19:628–634. https://doi.org/10.1038/gim.2016.175 27906198 10.1038/gim.2016.175

[mgg3307-bib-0022] Mahadeo, K. M. , N. Diop‐Bove , S. I. Ramirez , C. L. Cadilla , E. Rivera , M. Martin , et al. 2011 Prevalence of a loss‐of‐function mutation in the proton‐coupled folate transporter gene (PCFT‐SLC46A1) causing hereditary folate malabsorption in Puerto Rico. J. Pediatr. 159:623–627.e1. 21489556 10.1016/j.jpeds.2011.03.005 PMC3935241

[mgg3307-bib-0023] Olopade, O. I. , J. D. Fackenthal , G. Dunston , M. A. Tainsky , F. Collins , and C. Whitfield‐Broome . 2003 Breast cancer genetics in African Americans. Cancer 97:236–245. 12491487 10.1002/cncr.11019

[mgg3307-bib-0024] Prabhu, S. , and I. Pe'er . 2009 Overlapping pools for high‐throughput targeted resequencing. Genome Res. 19:1254–1261. 19447964 10.1101/gr.088559.108 PMC2704440

[mgg3307-bib-0025] Qiu, A. , M. Jansen , A. Sakaris , S. H. Min , S. Chattopadhyay , E. Tsai , et al. 2006 Identification of an intestinal folate transporter and the molecular basis for hereditary folate malabsorption. Cell 127:917–928. 17129779 10.1016/j.cell.2006.09.041

[mgg3307-bib-0026] Richards, S. , N. Aziz , S. Bale , D. Bick , S. Das , J. Gastier‐Foster , et al. 2015 Standards and guidelines for the interpretation of sequence variants: a joint consensus recommendation of the American College of Medical Genetics and Genomics and the Association for Molecular Pathology. Genet. Med. 17:405–424. 25741868 10.1038/gim.2015.30 PMC4544753

[mgg3307-bib-0027] Shah, K. B. , A. K. Mankad , A. Castano , O. O. Akinboboye , P. B. Duncan , I. V. Fergus , et al. 2016 Transthyretin cardiac amyloidosis in black Americans. Circ. Heart Fail. 9:e002558. 27188913 10.1161/CIRCHEARTFAILURE.115.002558 PMC4874558

[mgg3307-bib-0028] Sirugo, G. , I. M. Predazzi , J. Bartlett , A. Tacconelli , M. Walther , and S. M. Williams . 2014 G6PD A‐ deficiency and severe malaria in The Gambia: heterozygote advantage and possible homozygote disadvantage. Am. J. Trop. Med. Hyg. 90:856–859. 24615128 10.4269/ajtmh.13-0622 PMC4015578

[mgg3307-bib-0029] Strauss, K. A. , E. G. Puffenberger , and D. H. Morton . 2012 One community's effort to control genetic disease. Am. J. Public Health 102:1300–1306. 22594747 10.2105/AJPH.2011.300569 PMC3477994

[mgg3307-bib-0030] Suther, S. , and G. E. Kiros . 2009 Barriers to the use of genetic testing: a study of racial and ethnic disparities. Genet. Med. 11:655–662. 19752639 10.1097/GIM.0b013e3181ab22aa

[mgg3307-bib-0031] Tennessen, J. A. , A. W. Bigham , T. D. O'connor , W. Fu , E. E. Kenny , S. Gravel , et al. 2012 Evolution and functional impact of rare coding variation from deep sequencing of human exomes. Science 337:64–69. 22604720 10.1126/science.1219240 PMC3708544

[mgg3307-bib-0032] Watson, M. S. , M. Y. Mann , M. A. Lloyd‐Puryear , P. Rinaldo , and R. R. Howell . 2006 Newborn screening: toward a uniform screening panel and system. Genet. Med. 8(Suppl 1):1S–252S. 16783161 10.1097/01.gim.0000223891.82390.ad PMC3111605

